# X-ray Absorption Spectroscopy Study of Iron Site Manganese Substituted Yttrium Orthoferrite

**DOI:** 10.3390/molecules27217648

**Published:** 2022-11-07

**Authors:** Turghunjan Gholam, Hui-Qiong Wang

**Affiliations:** 1 Engineering Research Center of Micro-Nano Optoelectronic Materials and Devices, Ministry of Education, Fujian Key Laboratory of Semiconductor Materials and Applications, CI Center for OSED, and Department of Physics, Xiamen University, Xiamen 361005, China; 2 Department of Physics, Xiamen University Malaysia, Jalan Sunsuria, Bandar Sunsuria, Sepang 43900, Selangor, Malaysia; 3 Department of New Energy Science and Engineering, Xiamen University Malaysia, Jalan Sunsuria, Bandar Sunsuria, Sepang 43900, Selangor, Malaysia

**Keywords:** yttrium orthoferrite, hydrothermal method, X-ray absorption fine structure

## Abstract

In this work, manganese (Mn)-doped YFeO_3_, i.e., YFM*_x_*O powders with 0 ≤ *x* ≤ 0.1, was synthesized by a hydrothermal method to study the influences of doping on its structural, morphological, optical, magnetic, and local electrical properties. The experimental results show that all the samples exhibit an orthorhombic structure with space group *Pnma*. Refined structure parameters are presented. Morphology images show the shape evolution from layered to multilayered with increasing Mn content. Infrared spectra reveal the characteristic vibrations of the obtained YFM*_x_*O samples. From the magnetic study, an increased magnetic moment in the range of 0 ≤ *x* ≤ 0.075 is observed. The Fe and Y *K*-edge local structure studies indicate that the valency of Fe and Y is mainly found in the trivalent state, which also indicates that the substitution of Mn ions not only affects the nearest neighbor atomic shell of Fe but also affects the nearest neighbor’s local structure of Y atoms. Our results show that the addition of Mn exhibits an evident influence on the local structural and magnetic properties.

## 1. Introduction

The second-generation multiferroic materials, i.e., rare earth orthoferrites (RFeO_3_, R = rare earth ions), have been extensively studied for decades owing to their multiferroic, spin-switching, and magneto-optical properties [1,2,3]. Some other outstanding features of these materials, such as high domain wall velocity and the existence of Bloch lines [4], have promising applications in sensors, information storage, spintronics, etc. [5,6]. In most cases, RFeO_3_ is crystallized by the corner-linked FeO_6_ octahedral, forming a three-dimensional network in a centrosymmetric *Pbnm* (or *Pnma*) unit cell [7]. That is to say, the unit cell consists of four molecules with the R^3+^ cations located in the center and the Fe^3+^ ions are nearly octahedrally coordinated to six O^2−^ ions [8]. Unlike the first-generation multiferroics, RFeO_3_ not only combines antiferromagnetic and ferroelectric orders but also shows magnetoelectric coupling effects [9,10,11]. With the rise of RFeO_3_ materials in recent years, a member of them, yttrium orthoferrite (YFeO_3_, or YFO), has been most thoroughly investigated and has attracted much attention from the research community because of its magnetic, physical, and chemical properties due to the ionic and electronic defects as well as structure distortions [12]. YFO crystallizes in the ABO_3_ perovskite structure with the *Pnma* (D_2h_^16^) space group [13]. Despite the centrosymmetric nature, this material, with its low Curie temperature T*_C_* ~256 °C and high Néel temperature T*_N_* ~370 °C, can exhibit both ferroelectric and antiferromagnetic behaviors [14,15]. The multiferroic property of YFO is associated with the Fe spins [16]. As is commonly known, the doping technique is a very powerful way to improve the properties of a compound in many scientific areas. Cho et al. [17] studied the polarization properties and dielectric relaxation of YFe_0.8_Mn_0.2_O_3_ and they experimentally confirmed that there was no bulk polarization below room temperature. Xie et al. [18,19] observed a clear spin reorientation transition from Γ_4_ to Γ_1_, due to the partial substitution of manganese (Mn) at the iron (Fe) site of YFO. The work of Zhang et al. [20,21] showed that the Mn-doped YFO ceramics have spin glasslike behavior and distinct dielectric relaxation. In the report of Deka et al. [22,23], the single phase YFe*_x_*Mn_1−*x*_O_3_ samples show interesting spin orientation at low temperatures and the spin canting-induced weak ferromagnetism was also observed. Cao et al. [24] studied the Mn concentration-dependent conduction mechanism of pure YFO and YFe*_y_*Mn_1−*y*_O_3_ compounds. The doping of Mn for Fe effects on the structure and magnetic properties of YFO nanocrystal has been studied systematically by Shen et al. [25]. Mandal et al. [26] reported the observation of the magnetodielectric effect and the ferroelectricity under different temperatures in YFe_1-*x*_Mn*_x_*O_3_. The increase in antiferromagnetism and leakage current density of YFe_1-*x*_Mn*_x_*O_3_ compounds have been observed by Padmasree et al. [27]. A recent study of Mn-doped YFO by Suthar et al. [28] revealed that the dielectric loss increases with a doping concentration of Mn and the impedance was decreased with an increase in frequency, temperature, and Mn content.

Although there are many studies available for substitution of various ions on the Y/Fe site [29,30,31] or both the Y and Fe site [32,33] of YFO, the local electronic structure of Fe site Mn-doped YFO has rarely been investigated. The local electrical information of the sample is very important data for its applicability in practical use. To address this issue, we have studied the local structure properties of Fe site Mn-doped YFO powders, using an X-ray absorption fine structure (XAFS) spectroscopy technique. As is well known, XAFS has proven to be one of the best tools to probe the local electronic environment of each absorbing atom in simple or complex systems [34]. As a part of XAFS, X-ray absorption near edge structure (XANES) is very sensitive to the geometrical details of the absorbing atoms, e.g., formal oxidation state, specific symmetry, coordination chemistry, bond angles, etc. [35,36]. Another part of XAFS, extended X-ray absorption fine structure (EXAFS), is capable of determining atomic arrangements, which could provide reliable structural parameter information [37,38]. A fundamental step of the EXAFS analysis is the Fourier transformation, which is performed by fitting the *K* or *R* space, i.e., of all the atoms at a specific distance [39]. Usually, such a shell represents only one type of atom or different types of atoms at one distance [40]. If such an atomic shell consists of different elements, however, the Fourier transformation is unable to separate their waves. While one might solve this problem, the wavelet transformation is able to resolve the absorption signals in energy *E* space or wave vector *K* space [41]. In our work, both XANES and EXAFS were examined at the *K*-edges of Y and Fe. Wavelet transform was employed to the data analysis of EXAFS. We have also studied the morphological, optical, and magnetic properties of the target compound.

## 2. Results and Discussion

### 2.1. Crystal Structure and Rietveld Refinement

**Figure 1:**
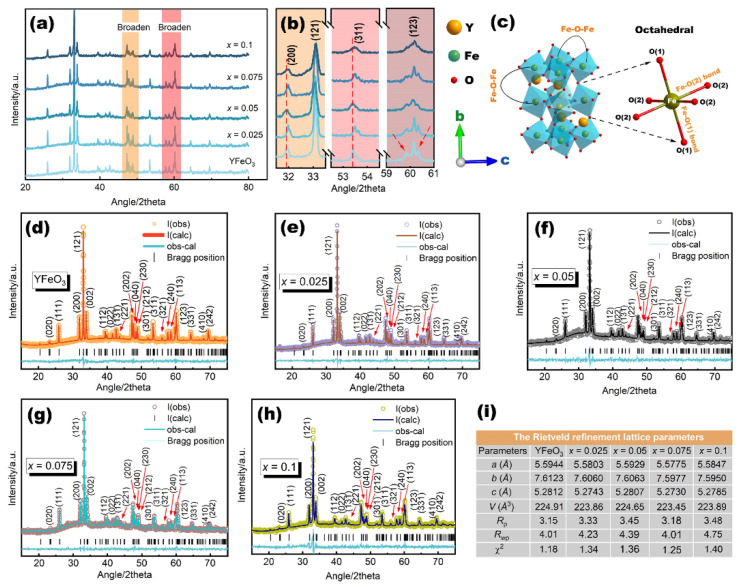
(**a**) XRD patterns of YFM*_x_*O (0 ≤ *x* ≤ 0.1); (**b**) enlargement patterns of the main peaks; (**c**) crystal structure of the YFM*_x_*O (*x* = 0.1); (**d**–**h**) Rietveld refinement profile of YFM*_x_*O; (**i**) lattice parameters of YFM*_x_*O.

Figure 1a presents the typical X-ray diffraction (XRD) patterns of YFe_1−*x*_Mn*_x_*O_3_ (YFM*x*O) powders with 0 ≤ *x* ≤ 0.1 at room temperature. The XRD diffraction peaks of all samples can be indexed as an orthorhombic structure with a space group of *Pnma* (ICDD File Card No. 01-086-0171) [42]. The diffraction patterns indicated a pure phase for all samples without any secondary phases such as Fe_2_O_3_, Y_2_O_3_, hexagonal *h*-YFO, etc., which indicated that all the prepared samples were highly pure. Our study shows that desired pure single-phase Mn-doped YFO compounds can be synthesized via the hydrothermal method. As is shown in Figure 1b, there is a slight shift of the major peaks, such as (200) and (311), to a bigger 2theta angle which becomes wider and weaker with the increase in Mn concentration. This shift in the diffraction angle is probably due to the difference in ion radius between the Mn and Fe ions. However, it is worth noting that the intensity of the (121), (202), (040), (230), (212), (311), and (123) diffraction peaks reduced and merged partially to form broadened peaks after the Mn concentration further increased, especially for the sample with *x* = 0.1. The XRD results show that the crystal retains its orthorhombic structure after Mn doping, but the quality of the crystal decreases, which is attributed to the presence of lattice distortions.

To quantify the structure in detail and determine the lattice parameters of the samples, an analysis of the XRD patterns by the Rietveld refinement was carried out using the *Pnma* space group in the orthorhombic unit cell. The Rietveld refined profile of YFM*_x_*O (0 ≤ *x* ≤ 0.1) powders are shown in Figure 1d–h. However, it is confirmed from the XRD results that the diffraction profiles belong to the orthoferrite structure for all samples. The structure parameters obtained from the Rietveld refinement have been used to sketch the crystal structure of the sample with *x* = 0.1, as shown in Figure 1c. Due to the low doping concentration, the crystal structure is almost identical to the pure YFO for each Mn-doped sample. In this structure, Y^3+^ is surrounded by 12 O^2−^ ions and Fe^3+^ is surrounded by six O^2−^ ions arranged in FeO_6_ octahedra. These O^2−^ ions connect two octahedral structures, which provide two types of super-exchange bonds, viz., two Fe-O(1)-Fe and four Fe-O(2)-Fe, respectively. Figure 1i shows the various structural parameters obtained from the Rietveld refinement. In this figure, the goodness of fitting χ^2^ is the conformity between an experimental result and theoretical expectation, and *R* values are useful indicators for the evaluation of a refinement. As is known, the Rietveld refinement results are reliable when the *R*_wp_ value is less than 10%. These parameters for each composition indicate that the quality of fitting is close to the best possible values, which means high reliability in our Rietveld refinement of YFM*_x_*O samples. According to refined values, lattice parameters of YFO are *a* = 5.5944 Å, *b* = 7.6123 Å, and *c* = 5.2812 Å and they are comparable to those reported in works of literature [43,44]. From Figure 1i, small changes observed in the values of lattice parameters and unit cell volume for the Mn-doped samples indicate that the substitution induces a small distortion in the crystal structure of YFO. The variations in the intensity of peaks and lattice parameters can also be attributed to the incorporation of the dopant in the crystal [45], which will be further illustrated by energy dispersive spectroscopy (EDS) results.

### 2.2. Morphological, Optical, and Magnetic Properties

**Figure 2:**
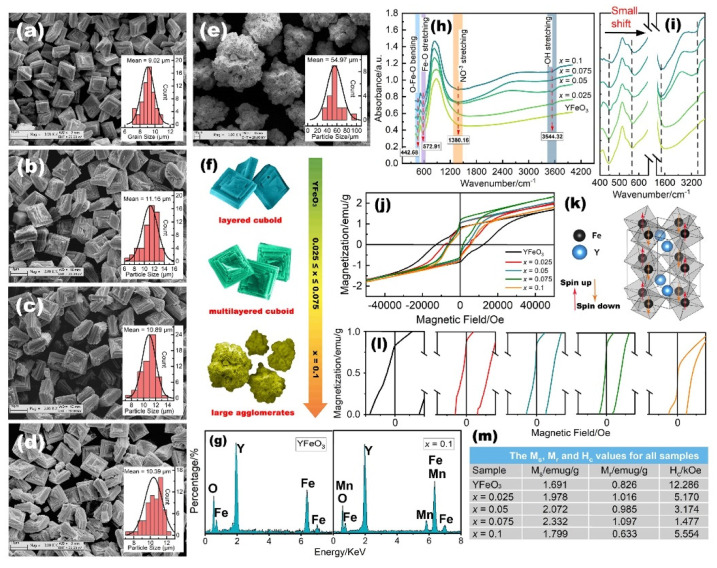
(**a**–**e**) SEM micrographs of YFM*_x_*O (0 ≤ *x* ≤ 0.1), the histograms in the insets are the grain size distributions; (**f**) schematic illustration of shape evolution; (**g**) EDS spectra of YFM*_x_*O (*x* = 0, 0.1); (**h**) FT-IR spectra of YFM*_x_*O; (**i**) selected part of the IR spectrum; (**j**) M-H loops of YFM*_x_*O; (**k**) magnetic moment in YFO structure; (**l**) zoomed-in view of M-H curves; (**m**) the M_s_, M_r_, and H_c_values of YFM*_x_*O.

The microstructural features of YFM*_x_*O (0 ≤ *x* ≤ 0.1) powders are investigated by the scanning electron microscope (SEM) micrographs, as shown in Figure 2a–e. The grain size distribution of these samples is shown in the insets of each micrograph. From these images, we can observe that the average grain size of pure YFO is about 9.02 μm, which is slightly increased to 11.16 μm, 10.89 μm, and 10.39 μm for *x* = 0.05 and *x* = 0.075, except for the sample with *x* = 0.1. It is seen that Mn ion substitution does not lead to a remarkable change in grain size (0 ≤ *x* ≤ 0.075), but it changes the morphology. It is observed that most of the pure YFO exhibits a layered cuboid shape. When the Mn content is up to *x* = 0.025, the multilayered cuboid is observed, and it is continuously layered with further Mn doping. In the hydrothermal crystallization processes of RFeO_3_, the addition of KOH could transfer R and Fe ions into amorphous hydroxides R(OH)_3_ and Fe(OH)_3_ in a very short time [9]. The formation of RFeO3 can be described by the chemical reactions, as follows: R^3+^ + OH^−^ = R (OH)_3_ (s); Fe^3+^ + OH^−^ = Fe (OH)_3_ (s). The transition metal or rare earth hydroxides usually form layered structures with ions inserted between the layers of metal hydroxide [46], which is in good agreement with the results observed by SEM. In contrast, when the Mn content reaches *x* = 0.1, the morphology changes to larger agglomerate shapes with varied grain sizes. The nucleation rate of the grains changed when the Mn concentration exceeded a certain value, and this has resulted in a different grain morphology. Figure 2f presents schematic illustrations of the morphological evolution of YFM*_x_*O powders. The crystal shapes of YFM*_x_*O powders are strongly dependent on the concentration of Mn, from which it can be concluded that YFO is a suitable compound to study the shape-dependent physical properties. Elemental compositions, as determined by EDS measurement, were performed on a selected area of pure YFO and sample with *x* = 0.1, as shown in Figure 2g. The obtained pure YFO reveals the existence of elements Y, Fe, and O. The corresponding EDS patterns of the *x* = 0.1 sample show the characteristic peaks belonging to the Y, Fe, Mn, and O, indicating the well-doped Mn in YFO. The spectra revealed that the molar ratios of existent elements are consistent with the chemical formula confirming the formation of target YFM*_x_*O compounds in the hydrothermal route.

The ideal non-distorted cubic perovskite structure ABO_3_ belongs to the space group of $O_{h}^{1}$ symmetry. Based on group theory, the active vibrations in the lattice consist of three modes, which can be attributed to the B-O stretching vibration of BO_6_ octahedra, the B-O bending vibration, and lattice vibration [47]. Figure 2h demonstrates the Fourier transform infrared (FT-IR) spectra of YFM*_x_*O (0 ≤ *x* ≤ 0.1) powder samples. At first sight, our experimental result shows only two active vibration bands in the IR spectra for all samples. This may be due to the substitution of Mn making the YFO structure more distorted. These two bands stem from the modes of Fe-O stretching vibration (572.91 cm^−1^) and O-Fe-O bending vibration (442.68 cm^−1^), respectively, being characteristics of the FeO_6_ octahedral in the perovskite compounds [48]. These vibration bands in IR spectra also confirm the formation of YFO in the hydrothermal method. The bands around 1380.16 cm^−1^ and 3544.32 cm^−1^ represent the absorption of NO^−3^ stretching vibrations from the small amount of trapped NO^−3^ ions in the YFO and -OH from ambient moisture, during the experiment, respectively. Based on further observation from the enlarged spectrum in Figure 2i, it can be seen that the absorption peaks slightly shifted to a larger energy side with increasing Mn concentration, which implies the increase in the covalence of the Fe-O and Fe-O-Fe bonds. A similar phenomenon has been observed in the work of Cao et al. [24]. However, the intensity of NO^−3^ and -OH bands slightly increased with increasing Mn content, indicating the incorporation of Mn ions at the Fe site of YFO. Except for these peaks discussed above, no additional peaks occurred. This is consistent with the result of the XRD measurement.

Figure 2j shows the obvious magnetization hysteresis (M-H) loops of YFM*_x_*O (0 ≤ *x* ≤ 0.1) samples measured at room temperature with a maximum magnetic field of 60 kOe. However, the loops of pure and doped samples are not saturated for an applied field up to 60 kOe. The hysteresis loop shows the typical canted antiferromagnetism as has been establish widely for YFO [49,50,51]. As is commonly known, the magnetic properties in rare earth orthoferrites originated from the super-exchange interaction of Fe^3+^-O^2−^-Fe^3+^, R^3+^-O^2−^-R^3+^, and R^3+^-O^2−^-Fe^3+^ [52]. In the orthorhombic YFO structure, each Fe^3+^ ion is surrounded by six O^2−^ ions to form FeO_6_ octahedra, and the O^2−^ ion is located in the union of two adjacent FeO_6_ octahedra playing a super-exchange interaction bond. Although the Fe^3+^ cation in the YFO structure is magnetic, the net magnetic moment is zero, since the octahedron site sublattice of Fe^3+^ cations is in an antiparallel position. A schematic illustration of magnetic moment in YFO is presented in Figure 2k. Typical plots of M-H loops of YFM*_x_*O (0 ≤ *x* ≤ 0.1) samples are shown in Figure 2i, on an expanded scale. It is observed that all samples exhibit canted antiferromagnetic behavior. In this structure, only the Fe^3+^ cations contribute on the magnetization, while the Y^3+^ cation is known as diamagnetic and has a zero magnetic moment, thus, the observed magnetization in our samples only comes from the Fe^3+^-O^2−^-Fe^3+^ super-exchange interaction. The saturation magnetization (M_s_), remnant magnetization (M_r_), and coercivity force (H_c_) values of the samples are listed in Figure 2m for comparison. Obviously, the magnetization of YFM*_x_*O is strongly related to the content of dopant Mn. It is observed that the M_s_, M_r_ values increase while the H_c_ values decrease for the sample with 0.025 ≤ *x* ≤ 0.075. This result can be attributed to several reasons as follows: Firstly, replacing Fe with Mn makes YFO more distorted, and increases the micro internal stress. Secondly, it could be due to the Fe^3+^-O^2−^-Fe^3+^ super-exchange interaction effect, which is the dominant magnetic interaction in this system. Thirdly, the magnetic contribution from the Mn cannot be ignored, which may be responsible for the increased magnetic moment with increasing Mn content. Although Mn ions can contribute to the magnetization, it is observed that the magnetic moment decreased when Mn concentration was up to *x* = 0.1. This may be due to the particle size effect, in which an increase in particle size leads to a decrease in magnetization, since the sample with *x* = 0.1 shows larger particle size than the other samples, as can be seen in the SEM images. Additionally, the substitution of Fe ions by an excessive amount of Mn ions may reduce the valence fluctuations and magnetization.

### 2.3. Fe K-Edge Local Electronic Structure

**Figure 3:**
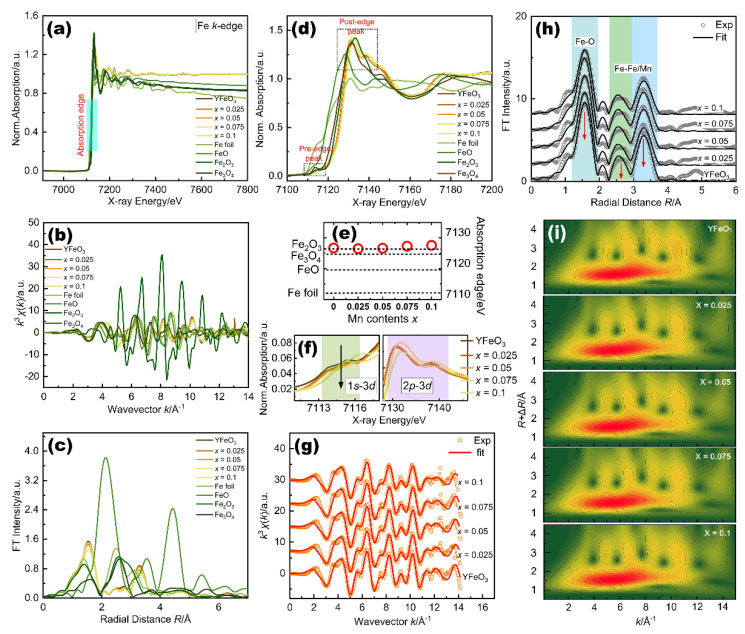
(**a**) Fe *K*-edge XANES full spectra of YFM*_x_*O (0 ≤ *x* ≤ 0.1) and reference samples; (**b**) EXAFS oscillations of YFM*_x_*O and reference samples; (**c**) Fourier transform EXAFS functions of YFM*_x_*O and reference samples; (**d**) magnified XANES spectrum; (**e**) composition dependence of the absorption edge *E*_o_. The dashed lines represent *E*_o_ of the reference samples; (**f**) enlarged XANES spectrum of the main peaks; (**g**) EXAFS spectra and best fits; (**h**) Fourier transform EXAFS data and best fits; (**i**) wavelet for the *k*^3^-weighted EXAFS signals; the data were offset for clarity.

Figure 3a shows the Fe K-edge XANES full spectra of YFM*_x_*O (0 ≤ *x* ≤ 0.1) with Fe foil, FeO, Fe_2_O_3_, and Fe_3_O_4_ as standard compounds. The XANES spectra of pure and doped samples show an analogous pattern to each other, which substantiates the fact that Mn ions have occupied the Fe sites of YFO. The Fourier transformation of the EXAFS is also given. EXAFS features could provide useful information on both the short-range and the long-range orders (i.e., in the first shell and higher shell than the second). Figure 3b shows the variation of the observed k^3^-weighted EXAFS oscillation of YFM*_x_*O (0 ≤ *x* ≤ 0.1) powders with standard compounds. The spectra of pure and Mn-doped samples are almost identical. The Fourier transforms of the k^3^-weighted EXAFS spectra of YFM*_x_*O (0 ≤ *x* ≤ 0.1) and reference samples are also shown in Figure 3c. The first and the second neighbor distributions in distance are easier to separate from the other shells in the Fourier transform. For the YFM*_x_*O (0 ≤ *x* ≤ 0.1) samples, there are some characteristic peaks in spectra: (1) There is a strong amplitude peak of about 1.54 Å, which corresponds to the Fe-O coordinate peak due to the first oxygen coordination sphere of Fe ions. (2) The second strong peak is about 3.29 Å, which is known as the Fe-Fe peak caused by the second rate of nearby metal ions. (3) The small intensity of other peaks is not yet clear. They are probably due to the multiple scattering processes in the first coordination shell. The magnified XANES spectra of YFM*_x_*O (0 ≤ *x* ≤ 0.1) and reference samples are presented in Figure 3d. The main peak in the spectrum consists of two parts: Pre-edge peak and post-edge peak. The pre-edge peak is usually related to quadrupole transition from 1*s* core state to 3*d* empty state, and contributions of dipolar transition originating from mixing of p with d orbitals, which is expected to be very weak for an Fe cation in an octahedral environment [53,54]. The invisible pre-edge peaks are observed in the spectra of both pure YFO and Mn-doped samples. The two post-edge peaks are attributed to the transfer of 2*p* electrons in the oxygen 2*p* band to the Fe 3*d* orbital by a shakedown process [55]. As seen in Figure 3e, for all the powder samples, the absorption edge energies were close to that of the reference sample Fe_2_O_3_. The valence state of Fe in Fe_2_O_3_ is 3+, which means the Fe atoms in all YFM*_x_*O samples have oxidation states of 3+. The enlargement of main peaks in the XANES spectrum of YFM*_x_*O (0 ≤ *x* ≤ 0.1) samples is shown in Figure 3f. It is well known that the pre-edge peak is a fingerprint of the octahedral coordination of Fe and the shifts of the pre-edge peak position towards higher energy with an increasing oxidation state [56]. In our samples, there is no pre-edge peak position shift, which further proves Fe is in a 3+ valance state in all the samples. Our spectra show that the intensity of the pre-edge peaks changes as a function of Mn content. From the enlarged XANES spectra in Figure 3f, it can be seen that, compared with the pure YFO, the intensity of the pre-edge peak is decreased for the Mn-doped sample with 0.025 < *x* < 0.075 and the intensity slightly increases for the *x* = 0.1 sample, which is due to the decrease in the symmetry of the Fe environment. A similar phenomenon has been observed in the other ABO_3_ perovskite system [57,58]. The decreasing intensity in the pre-edge peak was caused by the 1*s*–4*p* dipole-allowed transition while the increasing intensity indicates the enhancement of the 1*s*–3*d* electric dipole-forbidden transition. The decrease in pre-edge intensity caused by Mn substitution also indicates the increase in local structure distortion around the Fe ions [59]. The intensity of these two post-edge peaks first slightly increases and then decreases when *x* = 0.1 (see Figure 3f). This indicates that the 3*d*–4*p* transition and charge transfer from the O 2*p*–Fe 3*d* are enhanced with both low and high doping contents of Mn due to the loss of inversion octahedral symmetry of the oxygens around the Fe atoms [60]. These evolutions indicate that the local geometry and structure of Fe have changed.

In addition to the XANES data above, further analysis is carried out using the EXAFS oscillations of YFM*_x_*O (0 ≤ *x* ≤ 0.1) samples, and their theoretical fits are shown in Figure 3g. It shows that the experimental data and fitted data are well matched in the spectra, which means the spectra are of good quality. The error noise is observed above ca. 9 Å*^−^*^1^. Oscillations are still visible above ca. 12 Å*^−^*^1^, being less intense at the higher K. This phenomenon may be related to the less symmetric environment around Fe cations. Figure 3h shows the comparisons of the radial distribution functions between the experiment and fit for YFM*_x_*O (0 ≤ *x* ≤ 0.1) samples. As presented in Figure 3h, all samples show a strong peak at about 1.57 Å ascribed to the Fe-O scattering path in the first shell. There is a clear peak located at about 2.56 Å in the spectra. It could be recognized as a partial Fe atom, which coordinated with a Mn atom due to the electronic interaction. Moreover, the second dominant peak at about 3.30 Å refers to the Fe-Fe/Mn scattering path. Compared to the pure YFO, there is almost no shift of the peak position but the intensities of the Fe-O and Fe-Fe/Mn peaks are decreased as the Mn content increases. The reduction of the Fe-O peak intensity further indicates structural distortion, which is in line with the EXAFS analysis [61]. The intensities of the Fe-Fe/Mn peaks also have the same trend, which may be related to the lower photoelectron scattering amplitude of Fe due to the Mn addition. These changes in the spectrum may be due to the Mn atoms becoming closer at high concentration levels, leading to inhomogeneous distribution in the system, which further changes the local environment of Fe atoms. Moreover, the wavelet transformation of Fe K-edge EXAFS plots is also provided in Figure 3i. For both pure and Mn-doped samples, the signal from wavelet maxima near 5.8 Å^−1^ can be associated with F-O bonds. Maximum intensities at 6 Å*^−^*^1^ and 10.5 Å*^−^*^1^ are attributed to the Fe-Fe/Mn bonds, further confirming the existence of Fe-Fe/Mn bonds in the system. The maximum at 10.5 Å*^−^*^1^ is attributed to the Fe-Fe bond. These results are in good agreement with the EXAFS analysis.

### 2.4. Y K-Edge Local Electronic Structure

**Figure 4:**
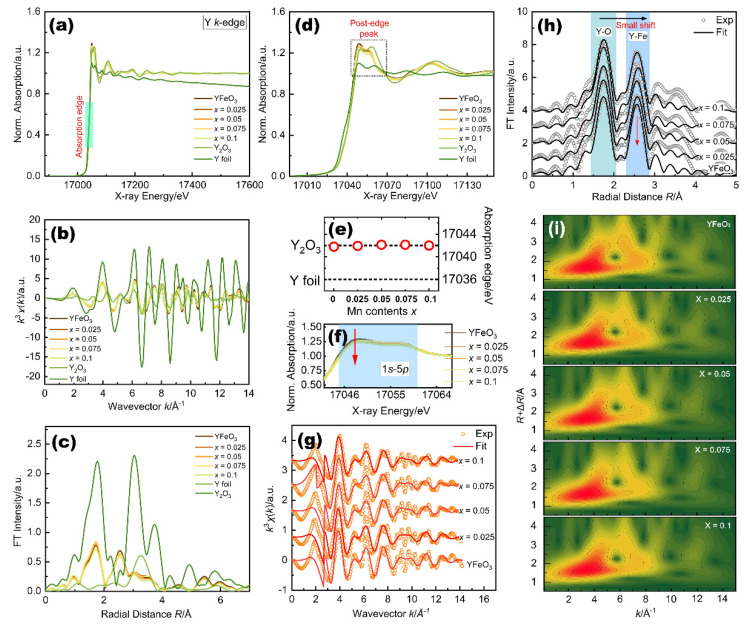
(**a**) Y *K*-edge XANES full spectra of YFM*_x_*O (0 ≤ *x* ≤ 0.1) and reference samples; (**b**) EXAFS oscillations of YFM*_x_*O and reference samples; (**c**) Fourier transform EXAFS functions of YFM*_x_*O and reference samples; (**d**) magnified XANES spectrum; (**e**) composition dependence of the absorption edge *E*_o_. The dashed lines represent *E*_o_ of the reference samples; (**f**) enlarged XANES spectrum of the main peaks; (**g**) EXAFS spectra and best fits; (**h**) Fourier transform EXAFS data and best fits; (**i**) wavelet for the *k*^3^-weighted EXAFS signals; the data were offset for clarity.

The normalized Y *K*-edge full XANES spectra of the studied YFM*_x_*O (0 ≤ *x* ≤ 0.1) samples, including the reference Y foil and Y_2_O_3_ compound, are shown in Figure 4a. It shows that the background correction and normalization result in a well-matched spectrum, which indicates the high quality of the spectra. The Y *K*-edge *k*^3^-weighted EXAFS curves of the YFM*_x_*O (0 ≤ *x* ≤ 0.1) with reference samples are given in Figure 4b. The Fourier transforms of the *k*^3^-weighted EXAFS functions of the YFM*_x_*O (0 ≤ *x* ≤ 0.1) powder samples with reference samples are shown in Figure 4c. From the figure, it can be seen that the first and the second neighbor distributions of Mn-doped samples are well separated from each other and other shells in the whole spectrum. The primary features are two dominant peaks and other small peaks for all samples distributed at different distances: (1) The first shell has an *R* of about 1.71 Å, corresponding to the Y-O peak caused by the scattering of oxygen anions from the nearest neighboring Y atomic shell. (2) The second shell with an *R* of about 2.54 Å corresponds to the Y-Y peak, which can be explained by the scattering of oxygen anions from the next nearest neighboring Y atomic shell. (3) The other small peaks are probably due to a large number of multiple scatterings in the first shell [62]. Figure 4d shows the enlarged XANES spectrum of YFM*_x_*O (0 ≤ *x* ≤ 0.1) powder samples. All the Mn-doped samples show nearly similar near-edge features, indicating a similar local structure around the Y ion in the first shell. The Y *K*-edge XANES spectra are similar to those of Fe *K*-edge XANES spectra without pre-edge peaks. The pre-edge peak is related to the 1*s* to 4*d* transition of Y, which partially allowed for the distortion of the octahedral, only when *p* orbitals were mixed with *d* orbitals. The fact that this transition is not observed indicates a small distortion of the octahedral symmetry [63,64]. The two main peaks can be observed in the spectrum (see Figure 4d), which could be due to the transition from the Y 1*s* state to the 5*p* state reflecting the density of unoccupied states in 4*d*–5*p* hybrid orbitals [65]. Generally, the shift of the absorption edge energy in XANES is sensitive to the oxidation state of Y in the material. The absorption edge position of the Y_2_O_3_ standard is close to that of the Mn-doped samples, as shown in Figure 4e. Since the valence state of Y is 3+ in Y_2_O_3_, this means the Y ions in our samples are in the 3+ valence state. From the examination of Figure 4d, it is clear that with increasing Mn concentration, there is no shift of the absorption edges in the whole series, but their intensity shows some difference. For better clarity, the enlarged post-edge peak is shown in Figure 4f, from which it can be seen that the intensity of the first strong post-edge peak decreases as Mn content increases. Such a change in intensity reflects a modulation in the electron density.

The EXAFS oscillation spectra and the best fit for YFM*_x_*O (0 ≤ *x* ≤ 0.1) samples are shown in Figure 4g. All the oscillations of pure and Mn-doped samples show similar patterns in the higher and lower *K* region. Figure 4h shows the corresponding Fourier transform EXAFS data and best fit for YFM*_x_*O (0 ≤ *x* ≤ 0.1) samples. The obvious strong peak located at 1.74 Å is assigned to the Y-O shell. Meanwhile, the second strong peak at about 2.56 Å corresponds to the Y-Y shell. The peak positions of Mn-doped samples shifted slightly to the higher *R* region, the intensities of which are affected by Mn substitution as well. Compared to pure YFO, the intensity of the Y-Y peak decreases with increasing Mn concentration. The reduction of the peak intensity represents the loss of short-range order in the system. These changes above indicated that the substitution of Mn ions not only affects the nearest neighbor atomic shell of Fe but also affects the nearest neighbor’s local structure of Y. Additionally, the wavelet transform EXAFS analysis was further performed to probe the Y species, as shown in Figure 4i. The wavelet plots of Y present two intensity maximums at about 3.8 Å^−1^ and 7.7 Å^−1^ that can be assigned to the Y-O and Y-Y coordination, coinciding with EXAFS analysis.

## 3. Materials and Methods

### 3.1. Sample Preparation

YFM*_x_*O powders with 0 ≤ *x* ≤ 0.1 were prepared by a hydrothermal synthesis process. High-purity yttrium nitrate (Y (NO_3_)_3_·6H_2_O, 99.9%), iron (III) nitrate (Fe (NO_3_)_3_·9H_2_O, 99.9%), manganese (II) chloride (MnCI_2_·4H_2_O, 99.9%), and potassium hydrate (KOH, 95%) were used as the starting materials. In the typical procedure, the Y (NO_3_)_3_·6H_2_O, Fe (NO_3_)_3_·9H_2_O, and MnCI_2_·4H_2_O were dissolved in 30 mL deionized water under magnetic stirring for 10 min until a clear solution was obtained. Then, 0.75 mol of solid KOH as the mineralizing agent was directly added into the mixture solution under magnetic stirring for 30 min. Those solutions were subsequently transferred into a Teflon-lined autoclave and heated for 72 h at 240 °C. After the hydrothermal reaction was complete, the resultant compounds were cooled to room temperature naturally. Finally, the products were washed several times with both ethanol and distilled water and then air-dried for 8 h at 80 °C.

### 3.2. Characterization

Phase purity and crystal structure were characterized by XRD on the Mac Science M18XHF22-SRA X-ray diffractometer using Cu Kα radiation (*λ* = 1.5406 Å). The crystal structure of YFO was examined employing Visualization of Electronics and Structural Analysis (VESTA) software. Rietveld refinement of the samples was performed using the GSAS-EXPGUI program. The SEM and EDS images were used to determine the shape, morphology, and composition of the samples using LEO1430VP equipment. The grain size was calculated using ImageJ software. Optical measurement was performed with FT-IR using the EQUINOX55 spectrometer. A vibrating sample magnetometer (VSM, MPMS-XL-7) was applied to measure the magnetic properties. XAFS spectra were recorded at the Beamline 1W2B of the Beijing Synchrotron Radiation Facility (BSRF), the Institute of High Energy Physics, China. Pellets prepared with boron nitride to optimize the thickness were used and put on tape for the detection of the whole experiment. XAFS spectra at the Fe and Y *K*-edges were collected in the transmission mode. Data elaboration has been performed using the ATHENA code of the IFEFFIT software package for analysis. The fitting part was performed on the filtered *k*^2^*χ*(*k*) signals using the ARTEMIS program [66]. *E*_o_ was defined as the maximum of the first derivative of the absorption edge. The atomic absorption data were a transition from *E* space to *K* space. A Fourier transform was performed to obtain a distribution function around the absorbing atom in radial distance *R* space. An isolated single shell *χ*(*k*) was obtained by the back transformation of the first shell signal from R space to K space. The *χ*(*k*)*k*^3^ of the EXAFS spectra was calculated in the interval *K* = 0.0 to 14.0 Å^−1^ and the Fourier transform of *χ*(*k*)*k*^3^ signals was taken from *R* = 0.00 to 6.0 Å, using a Hanning window function. The absorption profiles of iron foil (Fe foil), ferrous oxide (FeO), iron oxide (Fe_2_O_3_), ferriferrous oxide (Fe_3_O_4_), yttrium foil (Y foil), and yttrium oxide (Y_2_O_3_) were measured as references for valence state comparison. Wavelet transformation analysis has been performed using the HAMA Fortran software model.

## 4. Conclusions

In this work, the effect of Fe site Mn doping with 0 ≤ *x* ≤ 0.1 concentration on structural, morphological, optical, magnetic, and local electrical properties of YFO powders synthesized by using the hydrothermal method has been investigated. In the XRD patterns, the sharp and well-defined peaks show that all samples have an orthorhombic structure with space group *Pnma*. By using Rietveld fitting of the XRD profile, we could confirm the orthorhombic crystalline structure of YFO. As shown by SEM images, with increasing dopant concentration, the layered shape changes to a multilayered shape and a large particle size is observed when *x* = 0.1 with a larger agglomerate shape. The FT-IR spectra reveal the characteristic vibrations of the obtained samples. The magnetization has a close relationship with the Mn concentration. Magnetic hysteresis loop measurement showed the increased magnetic moment with increasing Mn concentrations in the range of 0 ≤ *x* ≤ 0.075. XAFS spectroscopy, including XANES and EXAFS with wavelet transform, has been used to investigate and obtain the structural information around Fe and Y atoms in the YFM*_x_*O samples. The results of XANES confirm the 3+ oxidation states of Y and Fe ions. Furthermore, the results from the EXAFS indicate that substitution of Mn ions not only affects the nearest neighbor atomic shell of Fe but also affects the nearest neighbor’s local structure of Y. Our results suggest that the Mn ions play an important role in the local structural and magnetic properties of YFO. Furthermore, the present work promotes the need for a better understanding of the local electronic structure in the YFO.

## Data Availability

Not applicable.
